# Enhancing Water-Splitting Efficiency Using a Zn/Sn-Doped PN Photoelectrode of Pseudocubic α-Fe_2_O_3_ Nanoparticles

**DOI:** 10.1186/s11671-020-03362-5

**Published:** 2020-06-15

**Authors:** Jie-Xiang Yang, Yongtao Meng, Chuan-Ming Tseng, Yan-Kai Huang, Tung-Ming Lin, Yang-Ming Wang, Jin-Pei Deng, Hsiang-Chiu Wu, Wei-Hsuan Hung

**Affiliations:** 1 Institute of Materials Science and Engineering, National Central University, Taoyuan, 32001 Taiwan; 2 Department of Materials Science and Engineering, Feng Chia University, Taichung, 40724 Taiwan; 3 College of Electrical Engineering and Automation, Shandong University of Science and Technology, Qingdao, 266590 China; 4 Department of Materials Engineering, Ming Chi University of Technology, New Taipei City, 24301 Taiwan; 5 Center for Plasma and Thin Film Technologies, Ming Chi University of Technology, New Taipei City, 24301 Taiwan; 6 Department of Chemistry, Tamkang University, New Taipei City, 25137 Taiwan; 7 Department of Mechanical Engineering, National Chung Cheng University, Chiayi, 621301 Taiwan; 8 High Entropy Materials Center, National Tsing Hua University, Hsinchu, 30013 Taiwan

**Keywords:** Pseudocubic α-Fe_2_O_3_, Water splitting, p-n junction, Solar energy

## Abstract

α-Phase hematite photoelectrodes can split water. This material is nontoxic, inexpensive, and chemically stable; its low energy gap of 2.3 eV absorbs light with wavelengths lower than 550 nm, accounting for approximately 30% of solar energy. Previously, we reported polyhedral pseudocubic α-Fe_2_O_3_ nanocrystals using a facile hydrothermal route to increase spatial charge separation, enhancing the photocurrent of photocatalytic activity in the water-splitting process. Here, we propose a p-n junction structure in the photoanode of pseudocubic α-Fe_2_O_3_ to improve short carrier diffusion length, which limits its photocatalytic efficiency. We dope Zn on top of an Fe_2_O_3_ photoanode to form a layer of p-type semiconductor material; Sn is doped from the FTO substrate to form a layer of n-type semiconductor material. The p-n junction, n-type Fe_2_O_3_:Sn and p-type Fe_2_O_3_:Zn, increase light absorption and charge separation caused by the internal electric field in the p-n junction.

## Introduction

**Fig. 1:**
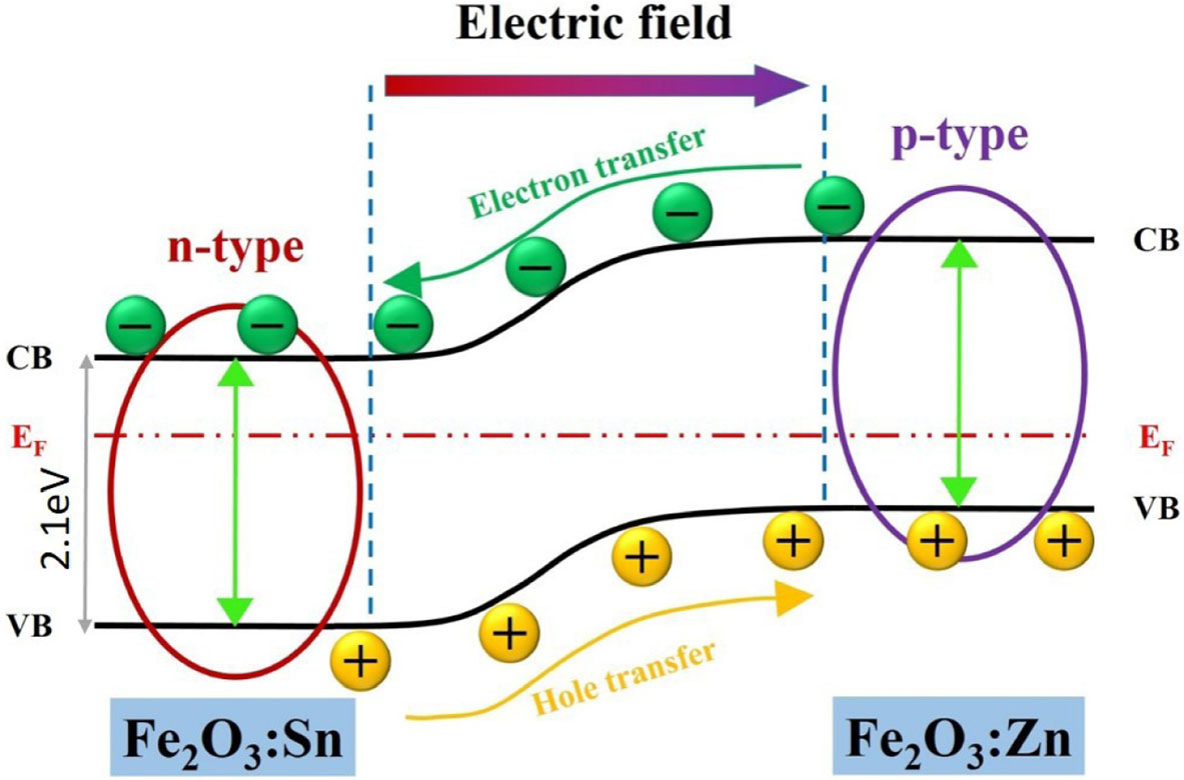
Concept of the p-n junction in a photoelectrode of polyhedral pseudocubic α-Fe_2_O_3_

To build a sustainable, renewable, and clean energy economy, solar-driven photoelectrochemical (PEC) water-splitting offers a promising route for effective solar fuel production. Most semiconductor materials possess reasonable sunlight absorption and conversion efficiencies as well as active catalytic properties; thus, they are strong candidates for photoelectrodes. Notably, hematite has attracted much attention because of its nontoxicity, high chemical stability, environmental compatibility, low cost, and low energy gap of 2.3 eV, which can effectively absorb wavelengths of less than 550 nm of visible light [1–5]. However, the PEC performance for water oxidation on α-Fe_2_O_3_ photoanodes [6, 7] is limited by their poor charge conductivity [8, 9] and mobility [10, 11], low absorption coefficient [8, 12], and rapid electron-hole recombination [13–15], which depresses the oxygen evolution reaction. To address these limitations, numerous approaches have focused on enhancing light absorption, the kinetics of the water oxidation reaction, and the charge-carrier collection efficiency through modifying electronic structural elements. For example, some studies have reported that introducing several types of ions into hematite could significantly improve the hematite carrier concentration and charge transfer rate at the surface [16–18]. In our previous study, we proposed facilitating the preferential migration of electrons and holes in semiconductors using differences in work functions at various crystal facets, which improved the spontaneous charge spatial separation during the water-splitting process [1, 19, 20]. In the present study, we sought to go further to improve the performance of water splitting based on the results of our previous study, combining the advantages of the existence of heteroions in photoanodes. Two types of ions, Zn and Sn, were incorporated into a layer of shaped controlled hematite cubes from the top and bottom, respectively, which also created concentration gradience differences in the two types of ions within the active layer of hematite (Fig. 1). In our previous study, Sn doping occurred spontaneously from the FTO substrate during the post-annealing process, and Zn doping was performed by spin-coating precursors of zinc acetate solution on the top surface of photoanodes and thermally reduced during post-annealing; this modified the flat-band potential at the semiconductor-electrolyte interface.

## Methods

Pseudocubic α-Fe_2_O_3_ nanocrystals were prepared through a hydrothermal route. In the synthesis of (012)-pseudocubic α-Fe_2_O_3_ nanocrystals, precursor Fe(acac)_3_ (2 mmol) and aqueous NaOH (0.6 M, 20 mL) were sequentially added to a solution of ethanol (20 mL) and DI-water (20 mL) with homogeneously vigorous stirring. Next, the mixed solution was placed in a Teflon-lined autoclave (100 mL) and maintained at 180 °C for 24 h. After being cooled to room temperature, the products were collected by centrifugation at 8000 rpm for 3 min and washed several times with n-hexane.

Subsequently, the products were ground into a powder and mixed with n-propyl ethanol (5 mL of n-propyl ethanol/0.1 g of powder) to obtain a suspension. In the doping process of Zn, we mixed zinc acetate and ethanol (0.1 g of zinc acetate + 2 mL of ethanol) to obtain zinc acetate solution. Finally, the pseudocubic α-Fe_2_O_3_ photoelectrodes were prepared using a spin-coating method and sintered at 450 °C for 10 h (heating rate = 2.5 °C/min) on the FTO substrate. In addition, Zn doping was prepared with a thermal diffusion method. We mixed zinc acetate and ethanol (0.1 g of zinc acetate + 2 ml of 99.5% ethanol) to obtain zinc acetate solution, which was then dropped 200 μL onto the pseudocubic α-Fe_2_O_3_ film. The active area of each sample was 1 × 1 cm^2^, and the mass loading of the Fe_2_O_3_ was approximately 0.2 mg. The prepared photoanode sintered at 450 °C for 10 h (heating rate = 2.5 °C/min) on the FTO substrate.

Characterizations of the pseudocubic Fe_2_O_3_ photoelectrode were performed using a field-emission scanning electron microscope (FE-SEM; S-4800, Hitachi) and high-resolution transmission electron microscope (HR-TEM; JEM-2100, JEOL). TEM samples were prepared by drop-casting an ethanol suspension of pseudocubic Fe_2_O_3_ NPs onto a copper grid. The composition and crystallinity of this Fe_2_O_3_ photoelectrode were determined using X-ray diffraction (XRD; D8 SSS Bruker). To study improvements to the separation of photoinduced charges, photoluminescence (PL) spectroscopy was performed to examine the recombination rate of photogenerated electron-hole pairs. The photon absorption properties of polyhedral α-Fe_2_O_3_ nanocrystals and their plasmon resonance were observed using ultraviolet-visible spectroscopy (UV-Vis; Lambda 650S, PerkinElmer). Photoelectrochemicals were measured using an electrochemical analyzer (CHI 6273E, CH Instruments) with a three-electrode electrochemical cell system in a darkroom (working electrode: hematite thin films, reference electrode: Ag/AgCl, counter electrode: carbon rod). The electrolyte was 1 M NaOH (pH = 14). In the photoelectrochemical measurement process, the light source was 532-nm laser irradiation (green solid laser, ALPHALAS) with a calibrated power density of 320 mW/mm^2^ with a spot size 1 mm in diameter. Hydrogen production was measured using gas chromatography (GC, China Chromatography GC1000TCD). Furthermore, the gas product was sampled every 20 min for 2 h.

## Results and Discussion

**Fig. 2:**
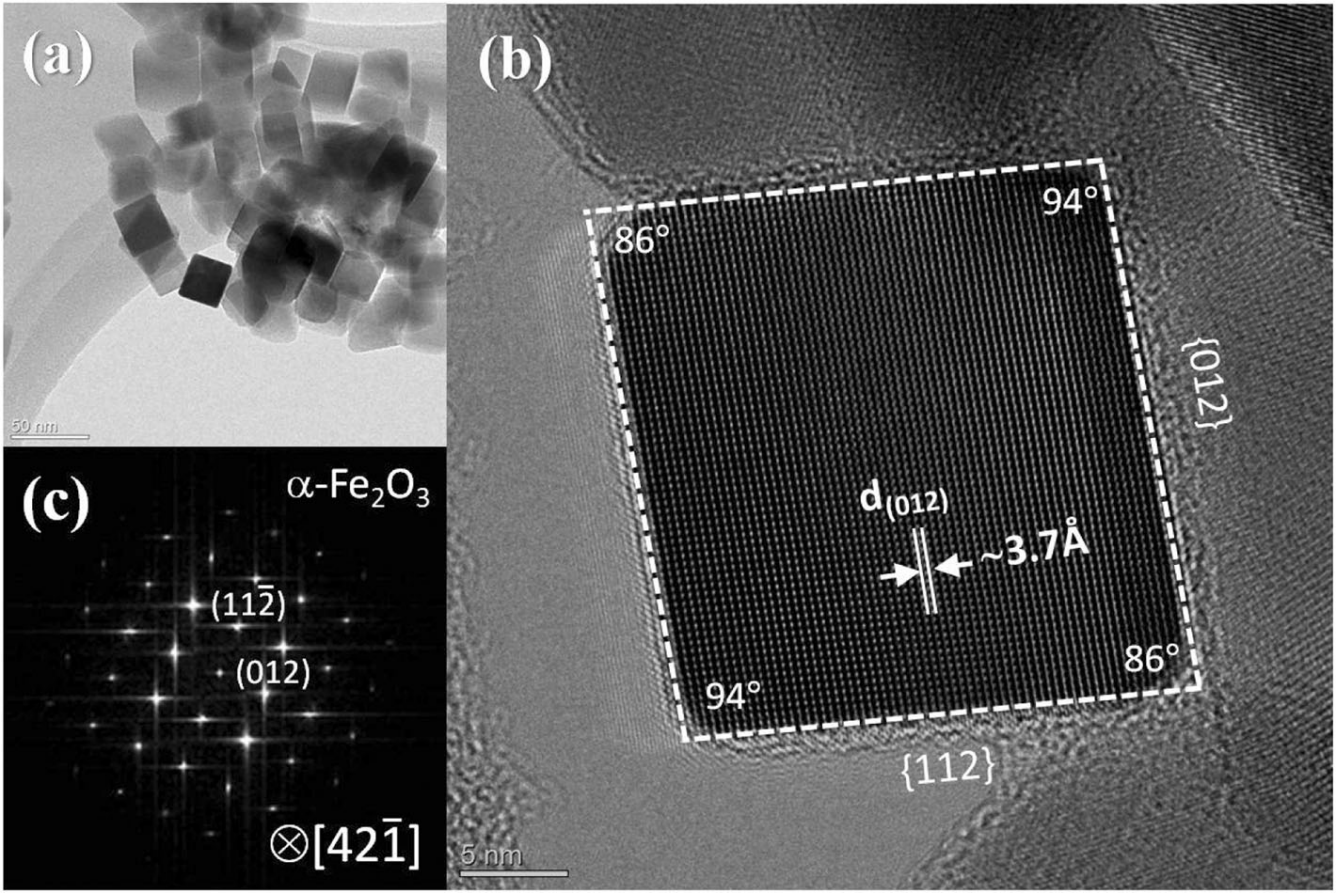
**a** TEM image of pseudocubic-Fe_2_O_3_ NPs. **b** High-resolution TEM image of a pseudocubic-Fe_2_O_3_ NP. **c** The FFT pattern in **b** reveals an α-Fe_2_O_3_ NP along its $\left[42\overline{1}\right]$ projection

Figure 2 presents TEM images of the α-Fe_2_O_3_, which indicate that the obtained particles possessed a pseudocubic shape and measured approximately 20 nm. The pseudocubic α-Fe_2_O_3_ consisted of (012) and (112) facets, and the crystallographic orientation was determined through the FFT pattern and high-resolution TEM images shown in Fig. 2b and c. These pseudocubic nanocrystals had an oblique parallelepiped morphology, where the dihedral angle between two adjacent facets was 86° or 94°. The FFT diffraction pattern shows that the (012) and (112) planes were nearest, and the interplanar distance was indicated as 3.7 Å along the [012] direction.

**Fig. 3:**
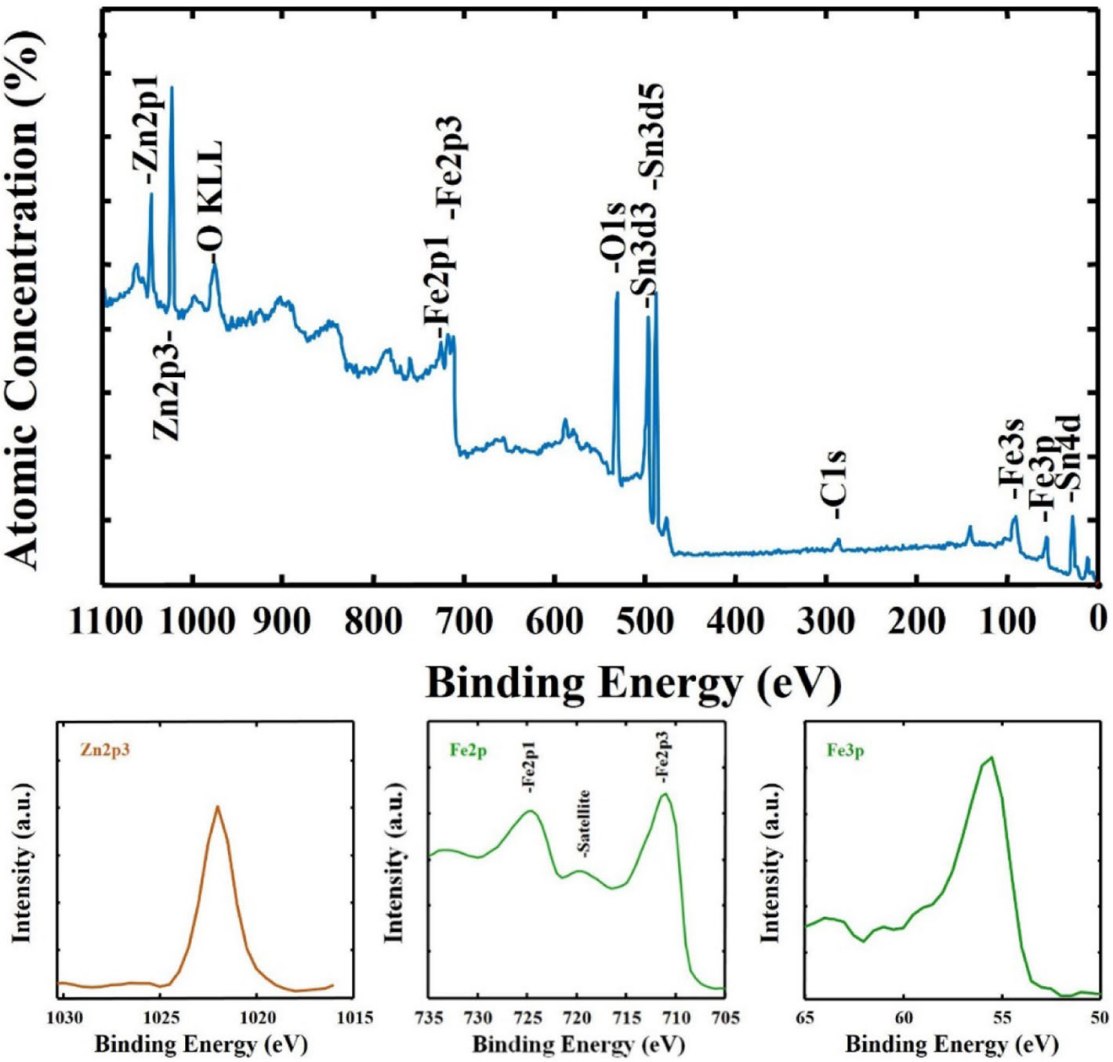
X-ray photoelectron spectroscopy (XPS) analysis of the Zn/Sn-doped p-n pseudocubic Fe_2_O_3_ photoelectrode: **a** survey XPS spectrum; **b** Fe 2*p*; and **c** Zn 2*p*

Figure 3 presents the XPS spectra of pseudocubic-Fe_2_O_3_:Zn/Sn for examining their chemical bonding state and electron bonding energy. In Fig. 3a, the presence of Zn in a-Fe_2_O_3_ was exhibited in the XPS spectrum, in which the peaks located at 1020.6 and 1044.1 eV were related to Zn 2p3/2 and Zn 2p1/2, respectively. In Fig. 3c, the high-resolution Zn 2p spectrum exhibits a pronounced peak centered at 1021.8 eV, corresponding to Zn 2p3/2, where the binding energy of Zn 2p3/2 is the typical value for ZnO; this suggested that the Zn dopant existed in the form of Zn^2+^. Zn was proved to be successfully doped within the Fe_2_O_3_. According to Fig. 3b, the XPS spectrum of Fe 2p3/2 and Fe2p1/2 in the Zn in a-Fe_2_O_3_ could be fitted as peaks at 710.7 and 724.3 eV, which was consistent with the binding energy of Fe^3+^ in the Fe_2_O_3_ origin.

**Fig. 4:**
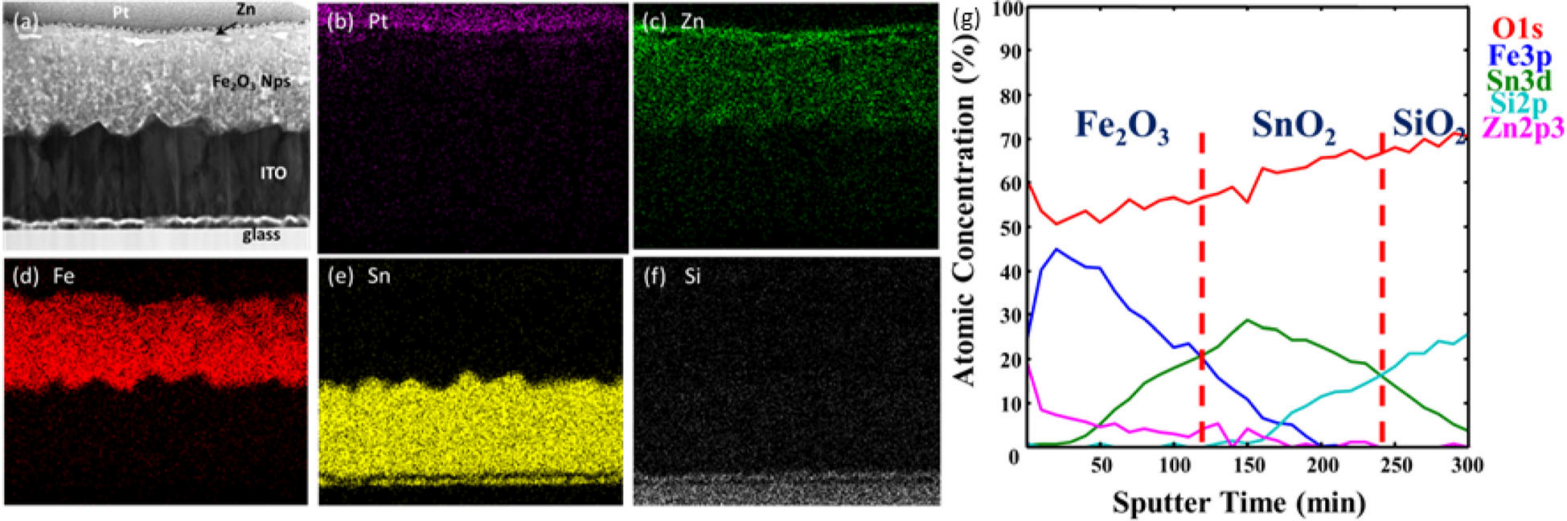
Cross-sectional imaging and chemical mapping of Zn/Sn-doped p-n pseudocubic Fe_2_O_3_ photoelectrode: **a**–**f** STEM images of the cross-section of an Zn/Sn-doped PN pseudocubic Fe_2_O_3_ photoelectrode. Note that the thin Pt layer seen in the image was deposited over the sample as a protection layer for the focused ion beam (FIB) milling step for cross-sectional sample preparation. **g** EDS mapping showing Zn, Fe, Sn, and Si elemental distributions respectively for the same sample as in **a**

Figure 4a–f shows a scanning transmission electron microscope with high-angle annular dark field (STEM-HAADF) cross-section micrograph of a Zn/Sn-doped PN pseudocubic Fe_2_O_3_ photoelectrode on an FTO-coated glass substrate. For protection purposes, Pt was coated onto the surface of the hematite film during the preparation of the TEM sample. Energy-dispersive spectroscopy (EDS) elemental maps of the Zn, Fe, Sn, and Si elemental distributions are shown in Fig. 4b–f, respectively. The pseudocubic Fe_2_O_3_ NPs could be observed to cover the FTO-coated substrate conformably. To examine the doping concentration distribution in depth, we performed an XPS depth scan. Figure 4 g depicts the atomic percentage (at%) of the elemental distributions as a function of sputter time for the pseudocubic-Fe_2_O_3_:Zn/Sn photoelectrode, along with a schematic representation of each layer. In this concentration depth profile, we observed the Zn 2p to exhibit the highest concentration at the top surface (approx. 20%), which decreased with sputter time. In addition, Sn diffusion from the FTO substrate was observed in our photoelectrode, which intercrossed with the Zn signal line at a sputter time of 50 min. The perfect spatial distribution of both Zn and Sn demonstrated a successful doping atom arrangement in the Zn/Sn-doped PN pseudocubic Fe_2_O_3_ photoelectrode. This result contributed toward an enhancement of the reaction photocurrent.

**Fig. 5:**
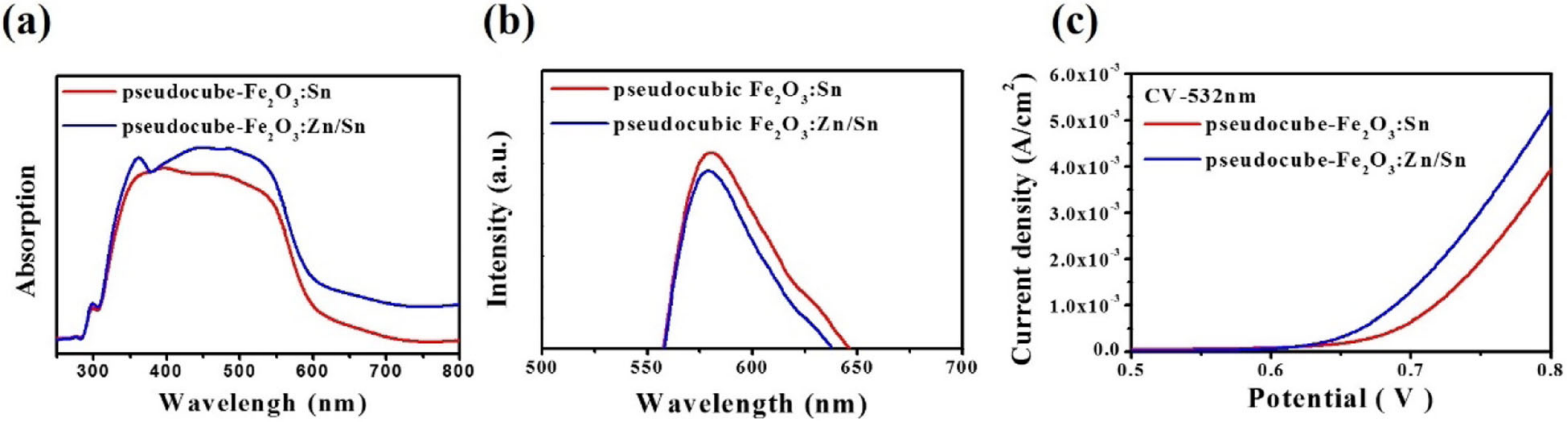
**a** Absorption spectrum of the photoelectrodes of Fe_2_O_3_:Sn and Fe_2_O_3_:Zn/Sn; **b** PL analysis of the Fe_2_O_3_:Sn and Fe_2_O_3_:Zn/Sn photoelectrodes; and **c** J-V scans collected for different doped Fe_2_O_3_

To identify the effect of pseudocubic Fe_2_O_3_:Sn with and without Zn doping, the absorption spectra of the Fe_2_O_3_:Sn and Fe_2_O_3_:Zn/Sn photoelectrodes were measured, as shown in Fig. 5a. The absorption spectrum of the Fe_2_O_3_:Zn/Sn (p-n junction) photoelectrode exhibited a stronger photon absorption crossover in the UV-to-visible light range. In addition, a small bump of an absorption peak appearing at 440 nm was observed; this was consistent with the absorption peak of the Zn NPs, which was because of the substitution between zinc and iron atoms [21–23]. Notably, a slight blue shift phenomenon was observed in the absorption spectrum after the Zn NPs were doped in the pseudocubic Fe_2_O_3_:Sn photoelectrode [24–26]. This phenomenon may be attributable to the Zn NP doping possibly raising the band gap of essential semiconductors [27–31]. Moreover, Mott-Schottky plot is performed for Zn/Sn -doped PN photoelectrode of pseudocubic α-Fe_2_O_3_ and have been characterized in Figure S1 in the supporting information. In the case of Zn/Sn-doped pseudocubic α-Fe_2_O_3_, it has been noted that both positive and negative slopes are observed, implying that the existence of the p and n type electronic behavior in our photoelectrode (shown in supporting information, Figure S2).

To further investigate the charge transfer of the photogenerated electron and hole pairs in pseudocubic Fe_2_O_3_:Zn/Sn, p-n junction system, this study used photoluminescence (PL) analysis, which could indicate the recombination of free charge carriers. Figure 5b shows the PL spectra of different samples with an excitation wavelength of 263 nm (4.71 eV). The pseudocubic Fe_2_O_3_:Zn/Sn displayed a lower PL intensity at approximately 580 nm, which was because of carrier diffusion between the p- and n-type semiconductor materials. This implied a decrease in electron and hole pair recombination, attributed to the p-n junction internal electric field.

Photocurrent responses were measured using a traditional three-electrode cell system. It was designed in a quartz cell, in which hematite thin films were used as the working electrode, Ag/AgCl as a reference, and a carbon rod as a counter electrode. The electrolyte was 1 M NaOH (pH = 14). In Fig. 5c, two different photoelectrodes with and without Zn doped, respectively, were tested under 532-nm laser irradiation. The pseudocubic Fe_2_O_3_:Sn and Fe_2_O_3_:Zn/Sn exhibited photocurrent densities of 4.1 × 10^−3^ and 5.3 × 10^−3^ A/cm^2^, respectively, at a bias voltage of 0.8 V. As expected, with superior performance in terms of the absorption spectrum and PL, the photocurrent-voltage (J-V) response of the pseudocubic Fe_2_O_3_:Zn/Sn (photocurrent density = 5.22 mA/cm^2^) was approximately 30% higher than that of the pseudocubic Fe_2_O_3_:Sn under 532-nm laser irradiation.

**Fig. 6:**
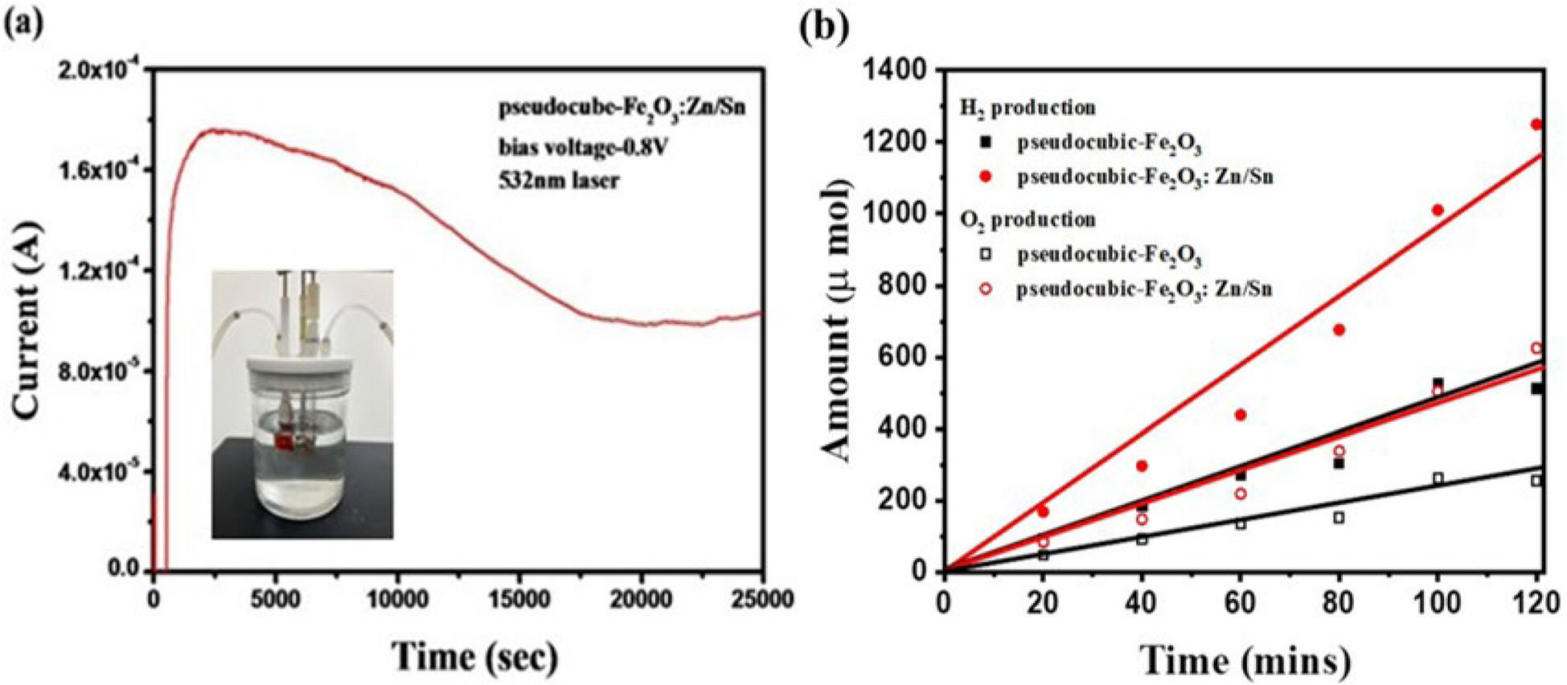
**a** Stability study of pseudocubic Fe_2_O_3_:Zn/Sn photoelectrodes (inset photo: our test system). **b** Production of H_2_ and O_2_ from pseudocubic Fe_2_O_3_:Zn/Sn photoelectrodes

The long-term stability of the Fe_2_O_3_:Zn/Sn photoelectrodes was tested under 532-nm laser irradiation for 7 h in Fig. 6a. The p-n junction system achieved a high light current response in a previous measurement. After irradiation for 7 h, the current response of the Fe_2_O_3_:Zn/Sn photoelectrode had only decayed by 35%, which confirmed that the Zn/Sn-doped PN pseudocubic Fe_2_O_3_ photoelectrode possessed strong photocurrent response stability. Finally, we examined H_2_ and O_2_ production to demonstrate a possible application of this high-performance PN photoelectrode; a comparison of H_2_ and O_2_ production from water-splitting was conducted and is presented in Fig. 6b for both the Fe_2_O_3_:Sn and Fe_2_O_3_:Zn/Sn samples. The Fe_2_O_3_:Zn/Sn photoelectrode generated approximately 1200 μmol of H_2_ and 520 μmol of O_2_ in 120 min, which were two times greater than those of pseudocubic Fe_2_O_3_:Sn.

## Conclusions

This study successfully demonstrated an enhanced charge spatial separation effect in pseudocubic Fe_2_O_3_:Zn/Sn photoelectrodes, which significantly improved performance in terms of photocurrent response and water-splitting gas products because of the built-in electric field. Furthermore, the Fe_2_O_3_:Zn/Sn photoelectrodes exhibited promising long-term stability, remaining at 70% magnitude of the initial photocurrent over 7 h of operation. This provides a significant water-splitting approach for sustainable energy conversion.

## Supplementary information

**Additional file 1: Figure S1**. Mott-Schottky plot of the Zn/Sn doped PN photoelectrode of pseudocubic α-Fe_2_O_3_. **Figure S2**. The comparison of XRD before/after operation.

## Data Availability

All data generated or analyzed during this study are included in this published article.

## Abbreviations

NP: Nanoparticle
PEC: Photoelectrochemical
FTO: Fluorine-doped tin oxide-coated glass
FE-SEM: Field-emission scanning electron microscope
HR-TEM: High-resolution transmission electron microscope
XRD: X-ray diffraction
PL: Photoluminescence
UV-Vis: Ultraviolet-visible spectroscopy
GC: Gas chromatography
XPS: X-ray photoelectron spectroscopy
EDS: Energy-dispersive spectroscopy
FIB: Focused ion beam
STEM-HAADF: High-angle annular dark field
