# Supplementary material for: Enhancing Water-Splitting Efficiency Using a Zn/Sn-Doped PN Photoelectrode of Pseudocubic α-Fe2O3 Nanoparticles
Source: Nanoscale Res Lett. 2020 Jun 15;15:130. doi: 10.1186/s11671-020-03362-5 (PMC7295917; doi:10.1186/s11671-020-03362-5)
Supplement: Supplementary file 1 — Additional file 1: Figure S1. Mott-Schottky plot of the Zn/Sn doped PN photoelectrode of pseudocubic α-Fe2O3. Figure S2. The comparison of XRD before/after operation. [file 11671_2020_3362_MOESM1_ESM.docx]

Enhancing Water-Splitting Efficiency Using a Zn/Sn Doped PN Photoelectrode of Pseudocubic α-Fe_2_O_3_ Nanoparticles

Jie-Xiang Yang^1, 5, #^, Yongtao Meng^4, #^, Chuan-Ming Tseng^2, 3,^ ***, Yan-Kai Huang^2^, Tung-Ming Lin^1, 5^, Yang-Ming Wang^1, 5^, Jin-Pei Deng^6^, Hsiang-Chiu Wu^7^, Wei-Hsuan Hung^1, 8,^ ***

_
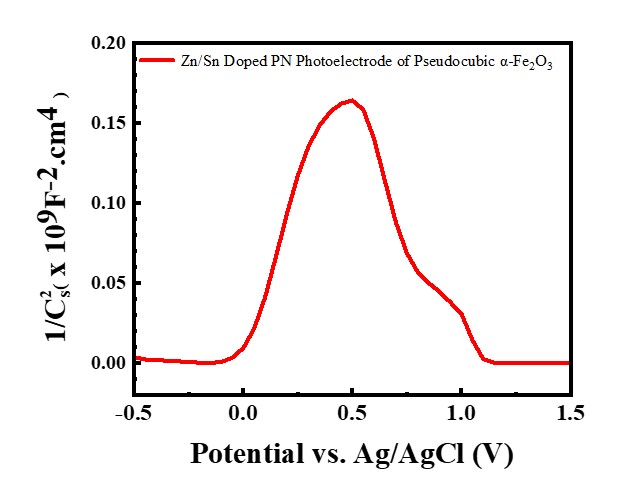
_

**Figure S1.** Mott-Schottky plot of the Zn/Sn doped PN photoelectrode of pseudocubic α-Fe_2_O_3_.


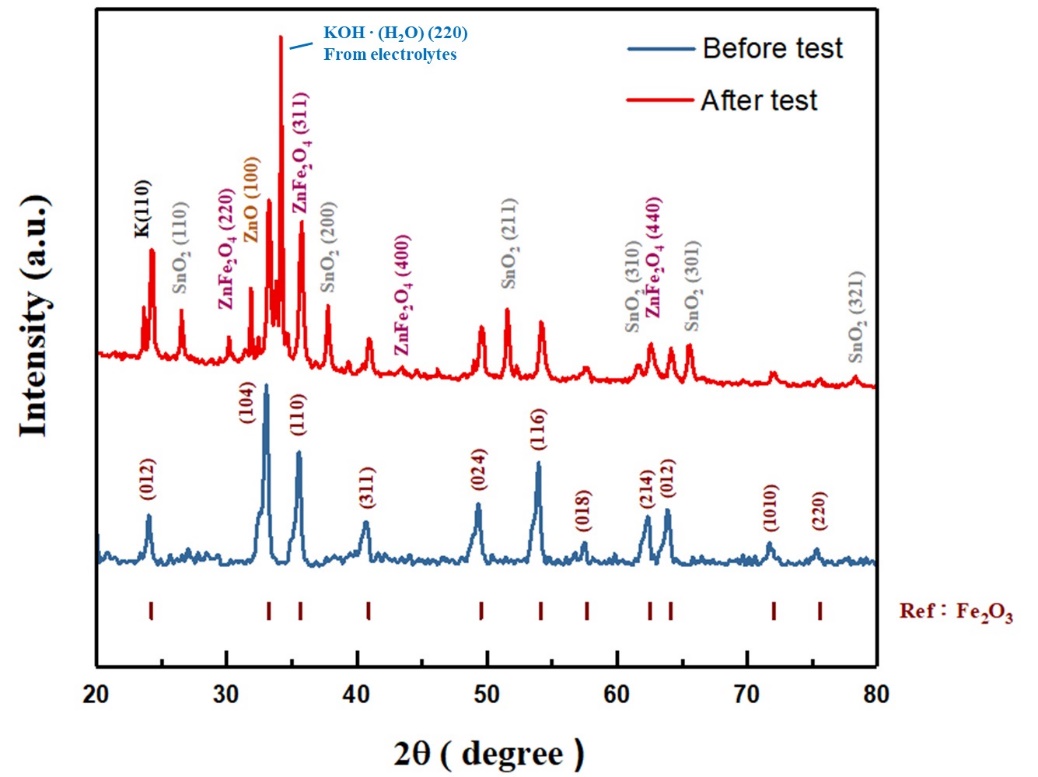


**Figure S2**. The comparison of XRD before/after operation.
